# Toxidermie médicamenteuse secondaire à l'acénocoumarol

**DOI:** 10.11604/pamj.2015.22.95.7809

**Published:** 2015-10-01

**Authors:** Naziha Khammassi, Nahed Kessentini

**Affiliations:** 1 Service de Médecine Interne, Hôpital Razi, Faculté de Médecine de Tunis, La Manouba 2010, Tunisie

**Keywords:** Toxidermie, iatrogénie, acénocoumarol, toxiderma, iatrogenic, acenocoumarol

## Image en medicine

Les toxidermies correspondent aux effets secondaires médicamenteux à expression cutanéo-muqueuses. Les médicaments les plus incriminés sont l'allopurinol, l'aminopénicilline, les céphalosporines, les antiépileptiques et les sulfamides antibactériens. L'exanthème maculo-papuleux secondaire à l'Acénocoumarol (Sintrom) est très rare. Le mécanisme est immuno-allergique. Patient B.H âgé de 44 ans, aux antécédents personnels de diabète de type 2, d'hypertension artérielle, d'accident vasculaire cérébrale ischémique en Mars 2015 gardant comme séquelle une hémiparésie gauche. En juillet 2015, il a présenté une douleur au niveau du mollet gauche. Une échographie doppler des membres inférieurs a été pratiquée montrant une thrombose veineuse profonde d'une veine soléaire externe gauche. Le patient a été mis sous Acénocoumarol (Sintrom) ½ comprimé/jour. Onze jours après, il a développé une éruption cutanée prurigineuse au niveau de faces antérieures et internes des avants bras ainsi qu'au niveau de la partie supérieure du dos évoluant dans un contexte d'apyrexie. A l'examen, il avait un visage bouffi, un œdème palpébrale et une éruption érythémato-papuleuse associée à des lésions de grattage au niveau des faces antérieures et internes des avants bras, au niveau de la partie supérieure du dos et au niveau du 1/3 inférieur des jambes sans atteinte des muqueuses. L'enquête de pharmacovigilance a conclu à l'imputabilité de l'Acénocoumarol, la conduite était d'arrêter ce dernier et d'introduire l'héparine. L’évolution a été marquée par la disparition des lésions au bout de 7 jours. D'autres diagnostics ont été évoqués mais non retenus à savoir les éruptions des maladies infectieuses, les maladies de système et la photosensibilité.

**Figure 1:**
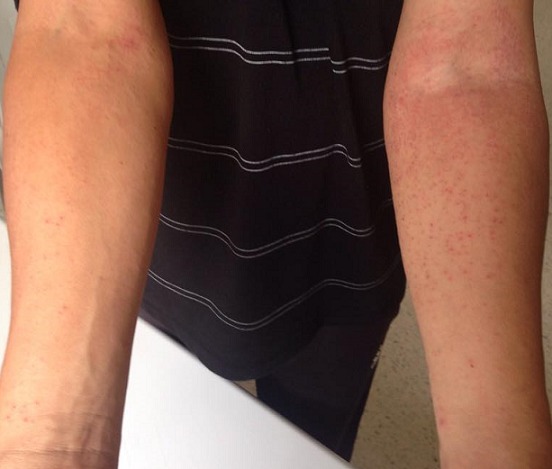
Éruption érythémato-papuleuse au niveau des faces antérieures des avants bras

